# Characterization of *SOD* and *GPX* Gene Families in the Soybeans in Response to Drought and Salinity Stresses

**DOI:** 10.3390/antiox11030460

**Published:** 2022-02-25

**Authors:** Muqadas Aleem, Saba Aleem, Iram Sharif, Zhiyi Wu, Maida Aleem, Ammara Tahir, Rana Muhammad Atif, Hafiza Masooma Naseer Cheema, Amir Shakeel, Sun Lei, Deyue Yu, Hui Wang, Prashant Kaushik, Mohammed Nasser Alyemeni, Javaid Akhter Bhat, Parvaiz Ahmad

**Affiliations:** 1 National Center for Soybean Improvement, State Key Laboratory of Crop Genetics and Germplasm Enhancement, Nanjing Agricultural University, Nanjing 210095, China; muqadasaleem@gmail.com (M.A.); 2018101110@njau.edu.cn (Z.W.); ammaratahirkahloon@gmail.com (A.T.); sunlei@njau.edu.cn (S.L.); 2 Center for Advanced Studies in Agriculture and Food Security (CAS-AFS), University of Agriculture, Faisalabad 38040, Pakistan; dratif@uaf.edu.pk; 3 Barani Agricultural Research Station, Fatehjang 43350, Pakistan; sabaaleem22@gmail.com; 4 Cotton Research Station, Ayub Agricultural Research Institute, Faisalabad 38040, Pakistan; iramsharif695@yahoo.com; 5 Department of Botany, University of Agriculture, Faisalabad 38040, Pakistan; maidaaleem357@gmail.com; 6 Department of Plant Breeding and Genetics, University of Agriculture, Faisalabad 38040, Pakistan; masooma@uaf.edu.pk (H.M.N.C.); dramirpbg@gmail.com (A.S.); 7 Instituto de Conservación y Mejora de la Agrodiversidad Valenciana, Universitat Politècnica de València, 46022 Valencia, Spain; prashantumri@gmail.com; 8 Botany and Microbiology Department, College of Science, King Saud University, Riyadh 12546, Saudi Arabia; mnyemeni@ksu.edu.sa; 9 International Genome Center, Jiangsu University, Zhenjiang 212013, China

**Keywords:** superoxide dismutase, glutathione peroxidase, *Glycine max*, *Glycine soja*, abiotic stress

## Abstract

Plant stresses causing accumulation of reactive oxidative species (ROS) are scavenged by effective antioxidant defense systems. Therefore, the present study performed genome-wide identification of superoxide dismutase (*SOD*) and glutathione peroxidase (*GPX*) gene families in cultivated and wild soybeans, and 11 other legume species. We identified a total of 101 and 95 genes of *SOD* and *GPX*, respectively, across thirteen legume species. The highest numbers of *SODs* and *GPXs* were identified in cultivated (*Glycine max*) and wild (*Glycine soja*). A comparative phylogenetic study revealed highest homology among the *SODs* and *GPXs* of cultivated and wild soybeans relative to other legumes. The exon/intron structure, motif and synteny blocks were conserved in both soybean species. According to Ka/Ks, purifying the selection played the major evolutionary role in these gene families, and segmental duplication are major driving force for *SODs* and *GPXs* expansion. In addition, the qRT-PCR analysis of the *G. max* and *G. soja SOD* and *GPX* genes revealed significant differential expression of these genes in response to oxidative, drought and salinity stresses in root tissue. In conclusion, our study provides new insights for the evolution of *SOD* and *GPX* gene families in legumes, and provides resources for further functional characterization of these genes for multiple stresses.

## 1. Introduction

Plants, being sessile, are often subjected to diverse environmental stress conditions such as abiotic and biotic stresses [1]. In this context, drought and salinity are the major abiotic stresses factors that plants face from germination to the harvesting stage [2]. Under these circumstances, limitation of CO_2_ intake due to stress-induced stomatal closure induces the photorespiratory production of reactive oxygen species (ROS). The main derivatives of ROS, including superoxide (O_2_^•−^), hydroxyl radical (^•^OH), per hydroxy radical (HO_2_^•)^, alkoxy radicals (RO^•^), hydrogen peroxide (H_2_O_2_) and singlet oxygen (^1^O_2_) are produced by the over-reduced photosynthetic electron transport chain. Under stress conditions, excess accumulation of ROS causes oxidative stress, which leads to extensive damage to DNA, protein and lipids, hence disturbing the normal metabolism and cellular functions [3,4,5].

In order to alleviate the ROS induced oxidative damage to plant tissues, plants have developed an antioxidant defense machinery to protect themselves from this damage [2]. This antioxidant defense system comprises both enzymatic and non-enzymatic ROS scavengers. For example, enzymes namely glutathione peroxidase (GPX), superoxide dismutase (SOD), catalase (CAT), ascorbate peroxidase (APX), monodehydroascorbate reductase, dehydro-ascorbate reductase, peroxiredoxin, glutathione reductase and glutathione S-transferase are enzymatic antioxidants involved in ROS detoxification [6]. The SOD provides the first line of defense against ROS toxicity in cells by catalyzing the conversion or dismutation of O_2_^●−^ radicals into H_2_O_2_ and molecular oxygen [3]. The SOD-mediated alleviation of the oxidative stress under different abiotic stresses has been reported in different plant species. For example, the transgenic rice overexpressing the *Mn-SOD* gene transformed from pea plant depicted improved resistance against drought stress, while in *B. napus* higher SOD activity was associated with salt tolerance [7,8]. Similarly, low expression of SOD enzyme under drought stress was found to be associated with more superoxide in *Sulla coronaria* (L.) Medik [9]. Increase in the expression of Cu-based SODs has been seen in *B. juncea*, while in *B. rapa* Mn- and Cu-based genes were found upregulated in response to heat stress [10]. The number of *SOD* genes has been reported to vary from seven to 29 in various species such as seven genes which were found in *Arabidopsis thaliana* and *Zea mays* [11]; 18 in *Gossypium hirsutum* [10,12]; 23 in *Triticum aestivum* [13]; and 29 in *Brassica juncea* [10].

To this end, the GPX is a non-heme containing peroxidase which plays a key role in the catalyzing of H_2_O_2_ along with other hydroperoxides into water and corresponding alcohols using reduced thioredoxin or other reducing equivalents as electron donors [14]. In various plant species, numerous genes encoding *GPX* have been identified and characterized. The effective role of *GPXs* has been recorded in protecting cells from oxidative damage by acting as efficient ROS scavengers under different abiotic stresses, e.g., salinity, heavy metal, cold and drought [15,16,17]. Salt tolerance ability of transgenic rice has been improved significantly by the overexpression of *NnGPX* gene, which is transformed from lotus [18]. In *Lotus japonicus*, the *GPXs* have been found as a major ROS scavenger in nodules as the overexpression of *LjGPX1* and *LjGPXs3* has been found to effectively provides protection against salt stress-induced oxidative damage especially in nodules [19]. Moreover, the *GPX* gene family has been recently characterized in the rapeseed, and they identified 25 *GPX* genes; moreover, their role in the multiple abiotic stress response and hormonal signaling has been documented [16]. Studies were also conducted to examine the changes in the levels of POD (glutathione) under salt and drought stresses in grass pea. Increase in POD (glutathione) activity was more exposed to drought in shoot while roots were under more salinity stress [20]. Genome-wide identification studies reported 13 *GPX* genes from *G. hirsutum*, and six in *Cucumis sativus* [21,22].

Legume (*Fabaceae*) is the third largest family of land plants with more than 20,000 species comprising of herbs, trees and shrubs [23,24]. Traditionally, based on the morphological characters, particularly floral ones, it has been further classified into three major subfamilies: *Papilionoideae*. *Mimosoideae* and *Caesalpinieae*. Furthermore, the *Papilionoideae* has been divided into four clades that consist mostly of economically important food and feed legumes [24]. Among them, soybean is the most important and cultivated legume with diverse uses such as its oil and protein consumption for humans and livestock, as well as feedstock for biofuel production. Soybean growth and development is badly affected by multiple abiotic stresses, i.e., drought, salinity and high temperature. The genus *Glycine* consists of two subgenera *Glycine wild* and *Soja*, and both these genera contain 28 species. Among these 28 species, the two annual species viz., *G. soja* (wild) and *G. max* (cultivated) are mostly consumed as food or feed either directly or indirectly. Wild soybean harbors precious genetic resources and an extraordinarily important gene pool responsible for resistance to abiotic (drought and high temperatures) and biotic stresses (disease and insect pest) [25].

By keeping the above into view, to date the *SOD* and *GPX* gene families have remained uncharacterized in cultivated and wild soybean as well as in other legumes. Hence, in the present study we carried out the genome-wide identification of *SOD* and *GPX* gene families in *G. soja* and *G. max* by employing bioinformatics approaches. Furthermore, the physicochemical properties, phylogenetic relationship, gene structure, protein motifs, promoter sequences, chromosome location, duplication and evolutionary sequence similarity were also analyzed for the identified *SOD* and *GPX* genes. Furthermore, expression analysis of randomly selected *GsoSODs* and *GsoGPXs* genes from wild soybean has been carried out to understand their response against drought, salinity and oxidative stresses.

## 2. Materials and Methods

### 2.1. Database Searches and Retrieval of SOD and GPX Genes

Genome sequences along with protein, gene sequences and annotation files of selected species were downloaded from their respective genomic databases are enlisted in Table 1. We filtered sequence files according to following criteria: (1) the longest transcript was selected to represent each locus, (2) coding sequences less than 150 bp were eliminated, and (3) the genes encoding the incomplete domain and truncated protein were discarded [26]. The sequences obtained after filtering were subjected to the Hidden Markov Model (HMM) of Pfam search of protein sequences to identify the protein domain family [27]. Furthermore, all the protein sequences were examined for the presence of *SODs* and the *GPXs* domain by the SMART tool [28]. For better understanding, the genes were named by following the approach; first three letters (i.e., *Gso*) representing the species name as *Glycine soja*, the next three letters (i.e., *SOD* or *GPX*) depicting the gene family, and the last two numbers represent as—1st digit represent chromosome number, and the second digit after the decimal represent gene number of gene family lying on a particular chromosome. For example, *GsoGPX2.4* refers the *GPX* gene located on chromosome 2 in *Glycine soja*, while 4 indicates the fourth *GPX* genes on chromosome 2.

### 2.2. Physicochemical Characteristics and Subcellular Localization

Physicochemical properties of SODs and GPXs proteins such as molecular weight, protein length and isoelectric point were checked by the ProtParam online tool [29]. Subcellular localization was predicted by using WegoLoc [30] and CELLO v.2.5 [31].

### 2.3. Multiple Sequence Alignment and Phylogenetic Analysis

Multiple sequence alignment was carried out by Clustal omega software [32], and the sequences with <50% identity were filtered out. Phylogenetic analysis was carried out to determine the evolutionary relationship of SOD and GPX proteins among 14 different species viz., *Glycine max*, *Glycine soja*, *Medicago truncatula, Lotus japonicus*, *Cicer arietinum*, Pisum *sativum*, Arachis *ipaensis*, *Arachis duranensis*, *Phaseolus vulgaris*, *Lupinus angustifolius*, *Vigna unguiculata*, *Vigna radiate*, *Vigna angularis* and *Cajanus cajan* along with model plant *Arabidopsis thaliana*. The phylogenetic tree was constructed in MEGA7 software [33], by employing the neighbor-joining method with 1000 bootstraps along with the poison model.

### 2.4. Gene Structure and Promoter Analysis

For the analysis of the intron–exon structure, the Gene Structure Display Server (GSDS) web tool was used by comparing the coding (CDS) and genomic sequences of each *SOD* and *GPX* genes [34]. MEME tool was used for the identification of conserved motifs. By submitting the protein sequences, we identified a maximum of 10 motifs with a motif width range from 6 to 50 [35].

In the *G. max* and *G. soja*, the 2000 bp upstream sequences from the translation start site of each *SOD* and *GPX* gene were downloaded and were used for promoter analysis. The promoter regions of each gene were screened for the *cis*-elements by using the PLANTCARE online tool [36].

### 2.5. Chromosomal Localization and Gene Duplication Analysis

Positional information of the *SOD* and *GPX* genes in *G. max*, *G. soja* and *Medicago truncatula* was obtained from the gene annotation file. The map diagram showing the location of *GPX* and *SOD* genes on chromosomes was drawn by using MapChart software [37]. Tandem duplications were defined as the homologous genes separated by five or fewer genes, while the genes separated by >5 genes or scattered on the different chromosome are known as segmental duplication. Gene duplication between *SOD* and *GPX* genes was identified by using the sequence demarcation tool SDTv1.2 [38]. The sequences with more than 90% similarity were considered as duplicated sequences.

### 2.6. Syntenic and Evolutionary Relationship

Sequence similarity between the SODs and GPXs protein sequence in *G. max*, *G. soja*, and *M. truncatula* was also carried by Circoletto software [39]. The *G. max* was used as a query against a database of *G. soja*, *Medicago truncatula* and *A. thaliana SOD* and *GPX* genes. The *E*-value was kept at 1 × 10^−10^, and it only shows the sequences that produced hits using percentage (%) identity (Blue = 70%, Green = 80%, and Orange = 90%) by keeping the rest of the parameters as default. Multi-sequence pairwise alignment of duplicated *SOD* and *GPX* genes was also carried out by the Clustal Omega and PAM weight matrix of MEGA7 software [33]. The subsequent alignments followed the synonymous (Ks) and non-synonymous (Ka) analysis for calculating the Ks and Ka substitution rates, respectively [40]. The ratio of Ka/Ks was calculated by the SNAP web tool to explore which type of codon selection activated during evolution. Probable time of the duplication event should also be calculated by using the formula T = Ks/2λ, whereas the value of λ = 6.1 × 10^−9^ was used for soybean [41].

### 2.7. Expression Pattern Detected by Transcriptome Data

To study the expression of *GmSOD* and *GmGPX* genes in different tissues and organs, transcriptome data extracted from a public soybean database were used to investigate the differential expression of *GmSOD* and *GmGPX* genes [42]. RNA-seq data of soybean *SOD* and *GPX* genes in different tissues were shown in Supplementary Table S1. A heatmap showing tissue-specific expression profiles was generated using the log_2_-transformed (FPKM + 1) values of *SOD* and *GPX* genes. Finally, visualization of the expression levels of *SOD* and *GPX* genes was accomplished by using the MeV 4.9.0 program (Anghel Saligny Nr 2 400609, Cluj-Napoca, Romania)

### 2.8. Plant Materials, Stress Treatments and Tissue Sampling

Representative and vigorous seeds of wild soybean viz., *Glycine soja* were used as a plant material for stress response investigations. These seeds were first disinfected by using 70% (*v/v*) ethanol, and then surface-sterilized for 4 h with sodium hypochlorite and hydrochloric acid (100 + 15 mL). The seeds of *G. soja* (genotype W05) were further germinated in plastic trays containing a mixture of soil and vermiculite at the ratio of 1:1. Seed germination was carried out in the incubator under controlled conditions by maintaining the proper environmental conditions as follows, i.e., the temperature was maintained at 25/23 °C, with a photoperiod of 16/8 h and 70% relative humidity (RH). At the second trifoliolate stage (V2), the soybean seedlings were transferred to the Hoagland solution, and the uniform length seedlings were planted in pots with 40 mL half strength Hoagland solution in each pot. The various abiotic stress treatments viz., drought, salinity and oxidative stresses were imposed when the second trifoliate leaves were fully expanded. For drought stress, the 15% PEG 6000 treatment were used in the Hoagland solution [43,44]. However, for salt treatment, the seedlings were subjected to 250 mM NaCl treatment in the Hoagland solution. For oxidative stress, the soybean seedlings were sprayed with the 10 mM H_2_O_2_. After exposure to each studied stress, the roots were sampled at 0 h, 3 h, 6 h and 12 h, respectively, from the 40–45-day-old seedlings. The completely randomized block design (CRBD) was used to plant the soybean seedlings, and three biological replicates were used for each stress treatment. Harvested root samples were immediately frozen in liquid nitrogen and stored at −80 ℃ until RNA extraction for quantitative real-time PCR (qRT-PCR).

### 2.9. RNA Isolation and Quantitative Real-Time PCR Analysis

To check the expression profiles of *SOD* and *GPX* genes in wild soybean, total RNA was isolated from the roots of soybean plants using the protocol suggested by the RNAprep Pure Plant Kit (Tiangen, China). Purity and concentration of the total RNA was determined by a Nanodrop ND-1000 spectrophotometer, and RNA integrity number (RIN) was measured using an Agilent Bioanalyzer 2100. The cDNA was synthesized using the Prime Script™ RT Reagent Kit (TaKaRa, Japan) according to the manufacturer’s instructions. Quantitative real-time PCR (qRT-PCR) was performed for each cDNA template using AceQ qPCR SYBR Green Master Mix (Vazyme, China) following the standard protocol. PCR amplification conditions were set as: 95 °C for 3 min; 35 cycles of 95 °C for 30 s, 58 °C for 20 s, and 72 °C for 20 s in a 20 µL reaction mixture. For qRT-PCR assays, three biological replications were used, and three measurements were performed on each replicate. The polymerase chain reaction (PCR) results were normalized using the *Ct* value corresponding to the soybean actin gene *GsoActin-11* (*Glysoja.18G049932*) as an internal control. Relative expression level for each gene was calculated by the 2^−ΔΔCt^ method [45]. All primers used were designed by the gene script web tool (Supplementary Table S2).

## 3. Results

### 3.1. Identification and Distribution of SOD and GPX Genes

By removing the non-targeted, overlapping or truncated protein sequences, we identified a total of 101 and 95 genes of *SOD* and *GPX*, respectively, in the 13 legume species (Supplementary Table S5). Genes from both families vary considerably among the 13 different species. For example, the number of *GPX* genes varied from 13 (*G. max* and *G. soja*) to three (*V. radiata*) (Supplementary Table S5), while as the *SOD* genes varied from 14 in *G. max* to three in *L. japonicus* and *P. vulgaris* (Supplementary Table S5). All these 101 SODS identified in 13 legume species contain conserved PF00080, PF00081 and PF02777 SOD domains. The Fe-SODs and Mn-SODs possess Iron/manganese superoxide dismutases, alpha-hairpin (Pfam: 00081), and C-terminal (Pfam: 02777) domains. The Cu/Zn-SODs had a copper/zinc superoxide dismutase domain (Pfam: 00080). In addition, the heavy metal domain (PF00403) was observed in the *GsoSOD5.1* and *GmaSOD5.1* (Supplementary Table S5). All the 95 *GPX* gene encoded proteins possess the Glutathione peroxidase (Pfam: PF00255) as a conserved domain (Supplementary Table S5).

The *SOD* genes encoded protein varied in the length from 149 to 388 amino acid residues (average length 227.93), has molecular weight ranging from 15.10 to 44.64 kDa and *pI* values varied from 4.73 to 8.97 23 in all the studied species. The protein length of GPX ranged from 114 to 327 amino acids (average length 194 amino acids), and the molecular weight from 12.74 to 160.39 kDa. An average weight of 23.58 kDa was recorded for GPX proteins. Moreover, the *pI* value was found to vary from 4.44 to 9.75, indicating a transition from an acidic to a basic nature of proteins.

In *G. max*, the length of SOD proteins ranged from 152 to 310 amino acids, while in *G. soja* it varied from 152 to 313 amino acids. Length of GPX proteins varied from 166 to 240 in *G. max*, and from 166 to 327 in *G. soja*. The *pI* values showed the acidic to basic nature of SODs (Table 1). Our results showed that the some of the gene pairs such as *GsoSOD2.1* vs. *GmaSOD2.1* and *GsoGPX5.1* vs. *GmaGPX5.1* from *G soja* and *G max* belonging to the same number of chromosomes were more evolutionary compared to each other in terms of protein weight, molecular weight (MW) and isoelectric point (PI). Information related to gene ID, rename ID, domain family and description, protein length, molecular weight, isoelectric point, and localization of *SOD* and *GPX* genes of 14 species are listed in Supplementary Table S5.

### 3.2. Phylogenetic Analysis of SOD and GPX Genes

**Figure 1:**
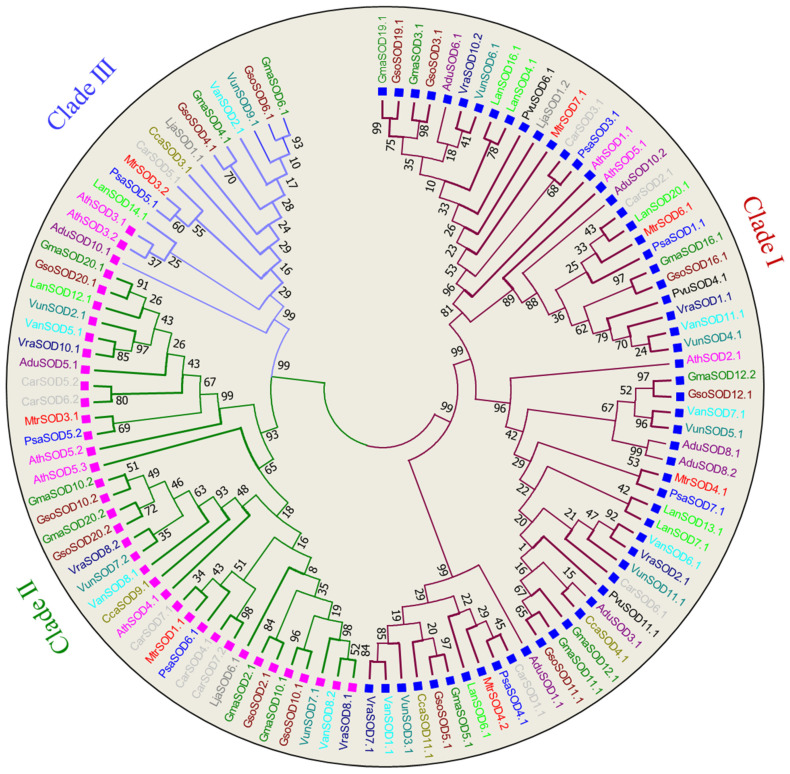
Phylogenic tree of SOD proteins. The tree was constructed in MEGA 7 by using the neighbor-joining method with 1000 bootstraps. Different clades of tree are marked with different colors: Clade I (Mahron), Clade II (Green), Clade III (Blue). The square shaped boxes represent the cellular localization: blue (cytoplasm) and pink (mitochondria).

Thirteen legume species along with model plant *A. thaliana* were selected to study the evolutionary history of *SOD* and *GPX* genes. Phylogenetic analysis of 109 SOD proteins was conducted to know the phylogenetic relationship among all the *SOD* genes of selected species. Based on tree topology, the SOD proteins of all species were categorized into three major groups viz., Cu/Zn-SODs (clade I), Fe-SODs (clade II) and Mn-SODs (clade III) (Figure 1). Clade I consisted of 59 Cu/Zn-SODs proteins, predicted to be localized in the cytoplasm, while Clade II clustered together 35 Fe-SODs that are localized in the chloroplast and Clade III was comprised of 15 Mn-SODs, suggested to be present in mitochondria. *SOD* genes belonging to Cu, Fe and Mn also varied between species. (Figure 1).

**Figure 2:**
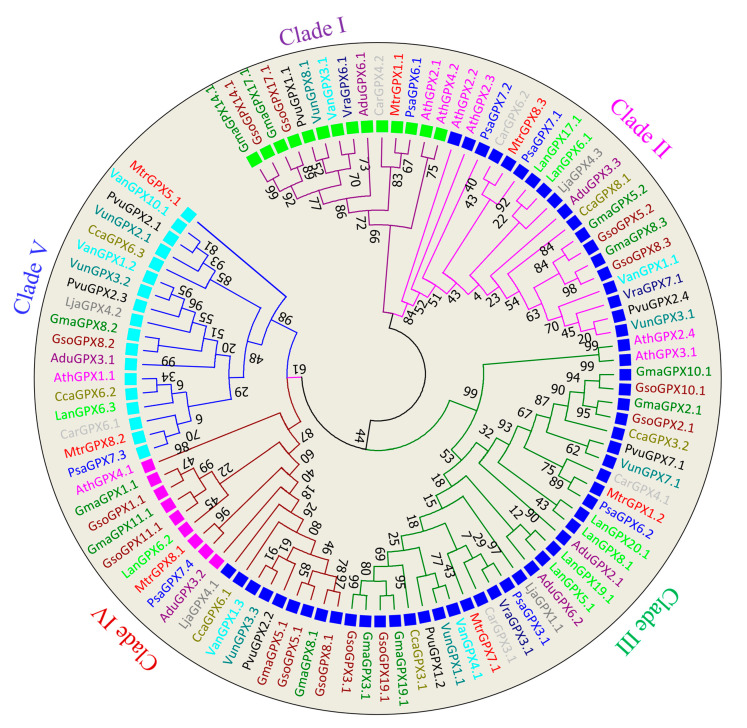
Phylogenic tree of GPX proteins. The tree was constructed in MEGA 7 by using the neighbor-joining method with 1000 bootstraps. Different clades of tree are marked with different colors of branch lines: Clade I (red), Clade II (pink), Clade III (green), Clade IV (mahroon) and Clade V (blue). The square shaped boxes represent the cellular localization: aqua (peroxisome), blue (cytoplasm) and green (chloroplast) and pink (mitochondria).

Phylogenetic analysis of GPXs members distributed them into five major clades, and each clade was represented with a different color of branch lines in Figure 2. Clade I consist of 14 members localized in the chloroplast; Clade II includes 19 members located in cytoplasm; Clade III is marked by thirty-one members with cytoplasmic localization, while Clade IV enclosed 18 members localized in both mitochondria and cytoplasm, such as mitochondrial (pink) and cytoplasm (blue). Clade V included 18 members with peroxisome localization. The clustering of the GPXs protein with different subcellular localization in the same group (clade II) depicts the gene duplication events leads to the formation of several GPX sequences. Tight clustering has been observed between *G. max* and *G. soja* genes compared other species, showing more similarity of *G. max GPX* genes with the *G. soja* genes compared to that of other legumes.

### 3.3. Exon–Intron Structures Analysis of SOD and GPX Genes in G. max, G. soja and Medicago truncatula

**Figure 3:**
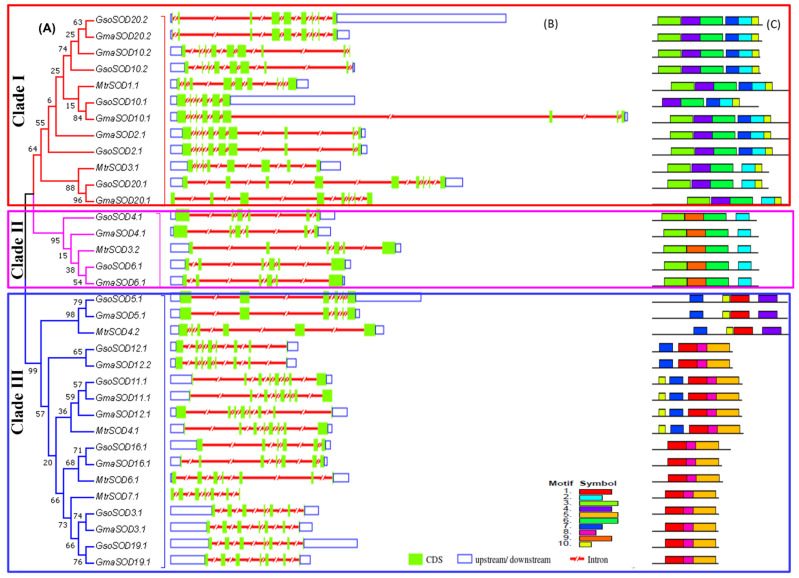
Phylogenetic relationship, exon–intron distribution/gene structure and conserved motifs in the *SOD* gene family in *G. max*, *G. soja* and *M. truncatula*. (**A**) The phylogenetic tree of the *SOD* gene family classified in to three clade: Clade I = Fe-SODs, Clade II = Mn-SODs, Clade III = Cu-SODs. (**B**) The untranslated region (UTRs), intron and exon distribution represented with the unfilled blue box, red shrinked line and green boxes, respectively. (**C**) Identification of the conserved motifs in *SOD* genes. Each motif is presented with a particular color.

**Figure 4:**
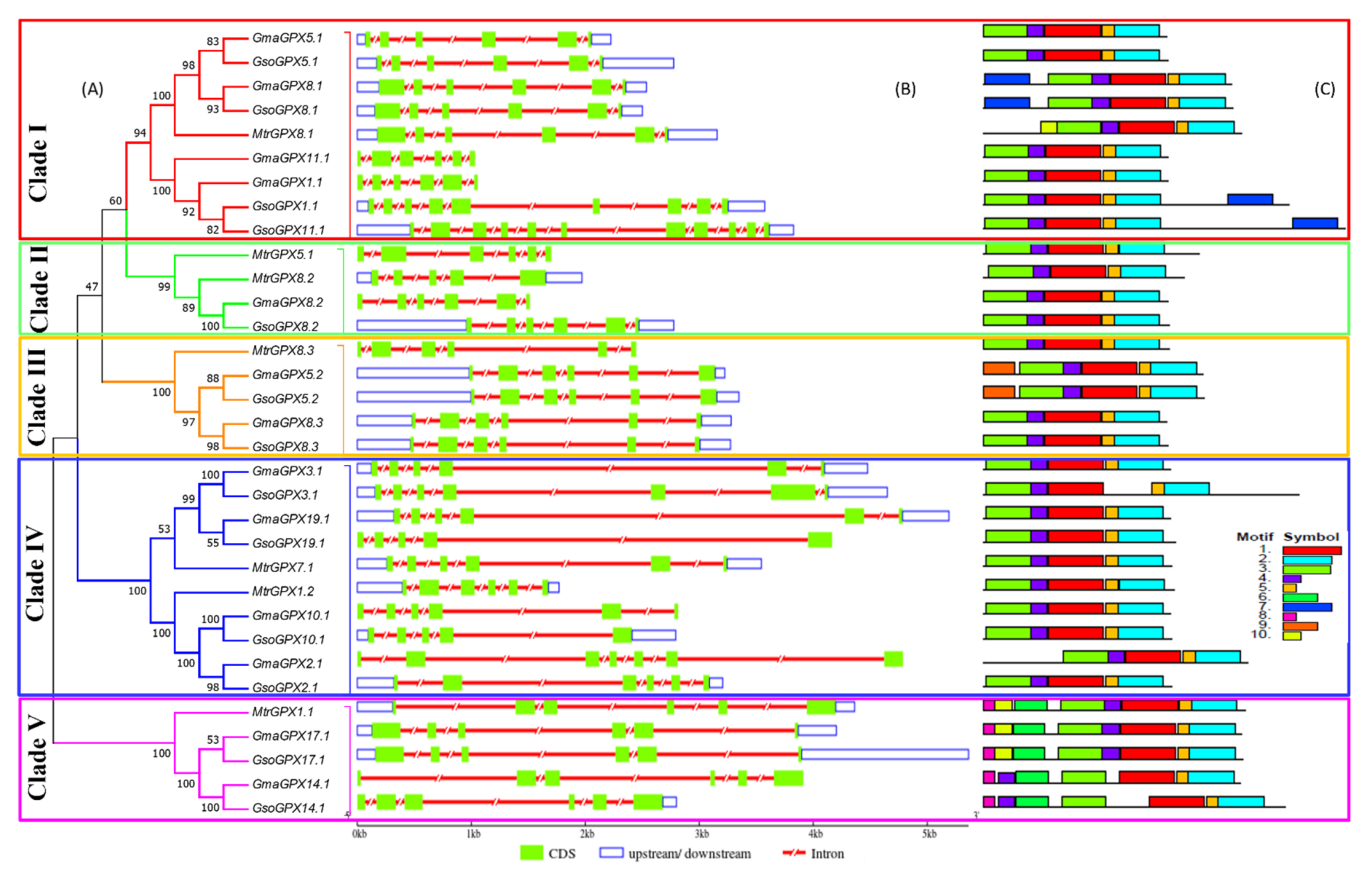
Phylogenetic relationship, exon–intron distribution/gene structure and conserved motifs in the *GPX* gene family in *G. max*, *G. soja* and *M. truncatula*. (**A**) The phylogenetic tree of *GPX* gene family classified into 5 clades represented with different colors of boxes on the phylogenetic tree (Clade I–V). (**B**) The untranslated region (UTRs), intron and exon distribution is represented with unfilled blue boxes, red shrinked lines and green boxes, respectively. (**C**) Identification of the conserved motifs in *GPX* genes. Each motif is presented with a particular color.

Gene structure diversity may guide understanding of the long-term evolution mechanism of multigene families. To obtain insight into structural features of *SOD* and *GPX* genes, a comparative analysis of exon–intron structure was executed for both soybean species along with *Medicago truncatula* as a model legume (Figure 3B and Figure 4B). Moreover, an unrooted phylogenetic tree of *SOD* and *GPX* genes was also constructed to view whether the exon/intron distribution pattern is consistent with the phylogenetic tree (Figure 3A and Figure 4A).

The *SOD* and *GPX* genes exhibited different exon–intron structures that were not in accordance with the phylogenetic clustering of these genes. The number of introns varied from five to seven in Cu/Zn SODs, five in Mn-SODs, and five to eight in Fe-SODs (Figure 3B). Species-wise, the number of introns varied from five to eight in all three species viz., *G. max*, *G. soja*, and *Medicago truncatula* for *SOD* genes, while it ranged from five to six in *G. max*, four to ten in *G. soja* and four to five in *Medicago truncatula* for *GPX* genes. For some of the genes, gene structure was conserved in wild and cultivated soybean, i.e., *GmaSOD2.1/GsoSOD2.1*, GmaGPX5.1/GsoGPX5.1, while some orthologue gene pairs showed different exon–intron structures such as *GmaSOD16.1/GsoSOD16.1* and *GmaGPX14.1/GsoGPX14.1*. However, more variation was seen in the exon–intron structure of soybean and *Medicago truncatula* *SOD* and *GPX* genes (Figure 3B and Figure 4B).

### 3.4. Conserved Motifs of SOD and GPX Genes in G. max, G. soja and Medicago truncatula

The presence of conserved motifs in *G. max*, *G. soja*, and *Medicago truncatula* was also explored in all the SOD and GPX proteins (Figure 3C and Figure 4C). In SODs, the conservation of motifs was more similar in each clade. Motifs 2, 3, 4, 6, and 10 were found in all FeSOD proteins except *GsoSOD10.1*, which did not possess motif 3. Mn-SOD proteins showed 2, 3, 6, and 9 as conserved motifs, while in all Cu/ZnSODs proteins, motif 1 remained conserved (Figure 3C). A total of ten conserved motifs were identified in *GPX* genes of both *Glycine max*, *Glycine soja*, and *Medicago truncatula* (Figure 4C). All of the *GPX* genes from *G. soja*, *G. max*, and *Medicago truncatula* carried 1–5 conserved motifs, which carried the GPX signature. Besides this, *GmaGPX8.1*, *GsoGPX8.1*, *GsoGPX1.1*, and *GsoGPX11.1* carried an additional motif 7 (Figure 4C). In group five, the *GPX* genes (*MtrGPX1.1*, *GmaGPX17.1*, *GsoGPX17.1*, *GmaGPX14.1*, and *GsoGPX14.1*) clustered together from *G. max*, *G. soja*, and *Medicago truncatula*, and they carried an additional motif 8. It is noteworthy that motif 9 was present in *G. max* and *G. soja* (*GmaGPX5.2* and *GsoGPX5.2*) and absent in *Medicago truncatula*. Overall the SOD and GPX motif structure was more conserved in *G. max*, *G.soja*, than *Medicago truncatula*, and some closely related genes shared a similar pattern of motif distribution. 

### 3.5. Promoter Analysis

The *SOD* and *GPX* genes play a vital role in response to environmental stresses. We analyzed the promoter of *G. max*, *G. soja* and *Medicago truncatula* SODs and GPXs by using 2000 bp upstream sequences to explore their putative *cis*-acting regulatory elements which play a key role in stress and hormone signaling. The results depicted that 77 types of putative *cis*-elements were present in the promoter region of all *SOD* genes of targeted species and seven were unknown elements (Supplementary Table S3).

The *cis*-acting regulatory elements involved in abiotic stress such as drought-responsive elements (as-1, DRE, MYC and MYB recognition sites), water-responsive element (MYB), and low temperature-responsive element (LTR) were found in the promoter region of *SOD* genes of soybean. Similarly to SODs, the abiotic stress-related *cis*-elements such as drought (MBS, as-1, and MYC), water (MYB), anoxia (ARE), and defense (TCT-motif), were identified in the promoter of *G. max*, *G. soja* and *Medicago truncatula*. Within the hormones regulating category, elements related to abscisic acid responsiveness (ABRE, ABRE2, ABRE3a, ABRE4 and CARE), gibberellin-responsive element (GARE-motif, P-box, and TATC-box), in salicylic acid responsiveness (TCA), auxin responsiveness (AuxRR-core, TCA, TGA, and TGA), methyl jasmonate (CGTCA-motif, TGACG-motif), circadian control (circadian) and ethylene-responsive element (ERE) were the most abundant in the promoter region of *SOD* genes, while in GPX the methyl jasmonate (TGACG-motif and CGTCA-motif), abscisic acid (ABRE), ethylene-responsive elements (ERE), salicylic acid responsiveness (TCA and TCA element), and auxin-responsive elements (TGA-element) were most common in both Soybean species (Supplementary Table S3). Furthermore, GPX from *G. max*, *G. soja* and *Medicago truncatula* harbored at least one type of hormone-regulating *cis*-element showing that GPXs are associated with the hormonal response. All 34 SODs in *G. max*, *G. soja*, and *Medicago truncatula* have conserved drought-responsive (MYC) and light-responsive cis-elements (Box-4), while *G. soja* also carried light responsive (G-box) and abscisic acid-responsive (ABRE) *cis*-elements in all *SOD* genes (Supplementary Table S3).

**Figure 5:**
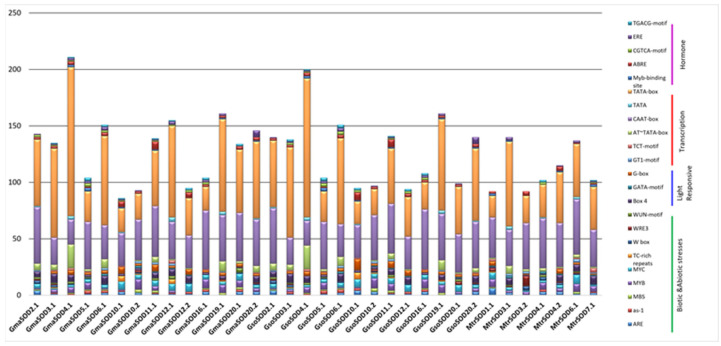
An overview of cis-regulatory elements in SOD family members of *G. max* and *G. soja*.

**Figure 6:**
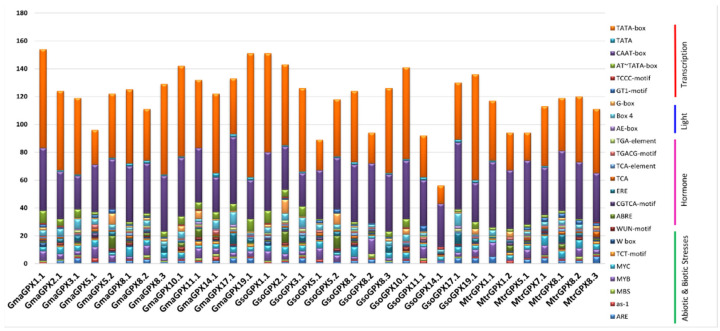
An overview of cis-regulatory elements in GPX family members of *G. max* and *G. soja*.

The putative 23 *cis*-elements (with a number greater than 15) identified in *SOD* and *GPX* genes promoter are presented in Figure 5 and Figure 6.

### 3.6. Chromosome Localization

**Figure 7:**
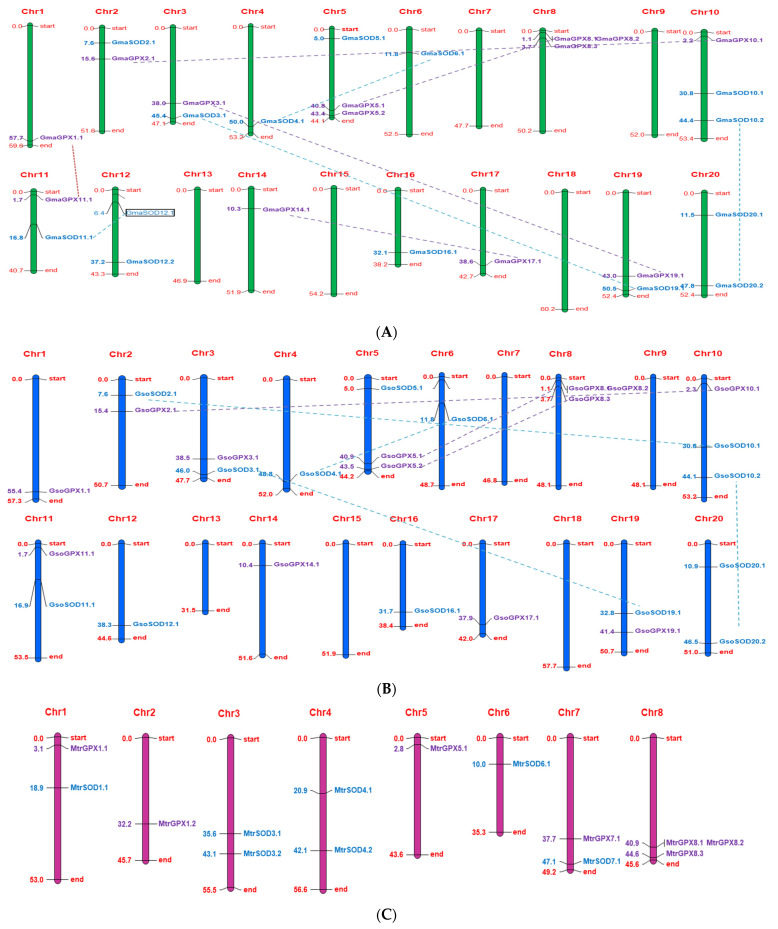
Chromosomal locations and gene duplication events of *SOD* and *GPX* genes of *G. max* (**A**), *G. soja* (**B**) and *M. truncatula* (**C**). The scale on the left side of chromosome is the position of the genes in megabases and on the right side of each chromosome gene names correspond to the approximate locations of each *SOD* and *GPX* gene. Furthermore, the segmentally duplicated genes are connected by dashed lines represented by the same color as *SOD* and *GPX* genes.

In *G. max*, out of total 20 pairs of chromosomes, the *SOD* genes were present only on 11 chromosomes. However, the eight chromosomes viz., 2, 3, 4, 5, 6, 11, 16 and 19 each carried one *SOD* gene, while chromosomes viz., 10, 12 and 20 possessed two SOD genes, and the rest of the chromosomes do not possess any *SOD* genes (Figure 7). In *G. soja*, the 13 *SOD* genes were mapped on 10 chromosomes (2, 3, 4, 6, 10, 11, 12, 16, 19, and 20), and among them chromosome 10 and 20 each contained two *SOD* genes similar to *G. max*, except chromosome 12. The occurrence of two genes on chromosome 12 in *G. max* compared to wild species may be due to gene duplication in *G. max* during domestication, as *GmaSOD12.1* and *GmaSOD12.2* shared a 100% similar sequence (Supplementary Table S4).

All the *GPX* genes were found to be unevenly distributed across different chromosomes of *G. max*, *G. soja*, and *M. truncatula.* Interestingly, *G.* *max* and *G. soja* not only shared an equal number of *GPX* genes (13 in number) but also depicted a similar distribution pattern of these genes in the genome (Figure 7). In both species, out of 20 chromosomes, all *GPXs* were organized across 10 different chromosomes. In *G. max* and *G. soja*, chromosome 1, 2, 3, 10, 11, 14, 17, and 19 carried one *GPX* gene, chromosome 5 possessed two, while chromosome 8 carried the maximum number of *GPX* genes (three). However, the rest of the chromosomes did not carry any *GPX* gene in both these species. In *M. truncatula*, seven *GPX* genes were arranged on five different chromosomes (1, 2, 5, 7, and 8), each carried one gene except chromosome 8 on which a cluster of three genes was found (Figure 7).

### 3.7. Gene Duplication

Due to genome duplication, tetrad, triplet, or paired homologous regions are accumulated in *Glycine*, and gene duplication events led to higher sequence similarity. Segmental duplication has been identified as a major driving force in the expansion of *SOD* and *GPX* genes in soybean. Three segmental duplications were identified in *G. max*: *GmaSOD12.1*/*GmaSOD11.1*, *GmaSOD4.1*/*GmaSOD6.1* and *GmaSOD20.2*/*GmaSOD10.2*, while four were found in *G. soja*: *GsoSOD3.1*/*GsoSOD19.1*, *GsoSOD4.1*/*GsoSOD6.1*, *GsoSOD10.2*/*GsoSOD20.2* and *GsoSOD10.1*/*GsoSOD2.1* in the *SOD* gene family, while a total of five segmental duplication events in *G. max*, and three in *G. soja* were observed in *GPX* genes (Table 2). Duplicate gene pairs are presented by dotted lines in Figure 7. No duplicated gene pairs were observed in *M. truncatula*.

Furthermore, through comparative study of *G. max* and *G. soja*, the 11 orthologue gene pairs have been identified in *SOD* genes, and five in *GPX* genes, that are 100% similar to each other in both species (Supplementary Table S4).

### 3.8. Divergence Rate of SOD Genes in G. max and G. soja

After gene duplication, the ratio of the nonsynonymous substitution rate (*Ka*) to the synonymous substitution rate (*Ks*) is employed to envisage the mechanism of gene divergence. The Ka/Ks ratio investigates the selection pressure in a gene pair. Moreover, the Ka/Ks < 1 represents the purifying selection, Ka/Ks = 1 shows the neutral selection, and Ka/Ks > 1 depicts positive selection [41]. We also calculated the Ka/Ks value of duplicated paralogous gene pairs in the *G. soja* and *G. max* genome. In *G. soja*, the Ka/Ks ratio of four duplicated *SOD* gene pairs ranged from 0.5 to 0.8, and it showed these duplicated gene pairs were subjected to purifying selection, while in *GPX* three duplicated gene pairs (Ka/Ks ratio from 0.079 to 0.197) were evolved mainly under strong purifying selection pressure, with less functional divergence after duplication (Table 2). In *G. max*, two gene pairs of both *SOD* and *GPX* genes were subject to purifying selection (0.7–0.8). One gene pair (*GmaGPX3.1*/*GmaGPX19.1*) showed only neutral selection (Ka/Ks = 0.970 ≈ 1), while, in the rest of the gene pairs of *SOD* and *GPX* genes, the Ka/Ks ratio was greater than 1, which seemed to have undergone strong positive selection (Table 2). Divergence time of these duplicated gene pairs varied from 0.00 to 10.46 Mya. After duplication, positive selection is regarded as one of the main forces for the evolution of new motifs or functions.

### 3.9. Relationships between GmaSODs with GsoSODs, MtrSODs and AthSODs

**Figure 8:**
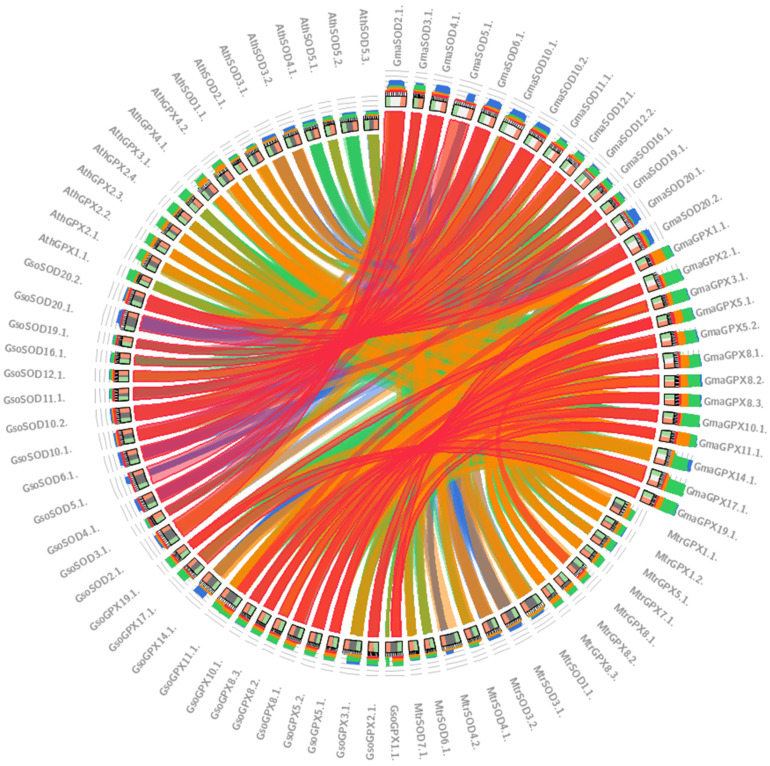
The synteny relationship between *SOD* and *GPX* gene families in *A. thialana*, *G. max*, *G. soja* and *M. truncatula* by using Circos tool. The blue, green, orange and red colors represent ≤50%, ≤70%, ≤90% and >90% identity, respectively.

Comparative genomics assists in revealing the evolutionary relationship among the different species. Synteny was visualized among *SOD* and *GPX* genes of the four species viz., *G. max*, *G. soja*, *Medicago truncatula* and *A. thaliana*. The SODs and GPXs belonging to *G. max* and *G. soja* are mostly connected via red lines showing more than 90% identity. As *G. soja* is considered as the progenitor of the *G. max*, the same thing was supported by our results where some antioxidants gene sequences of *G. max* were 100% identical to *G. soja* genes, whereas the majority showed more than 90% similarity (Figure 8).

### 3.10. Expression Profiling of SOD and GPX Genes in Different Soybean (Glycine max *L.*) Tissues

**Figure 9:**
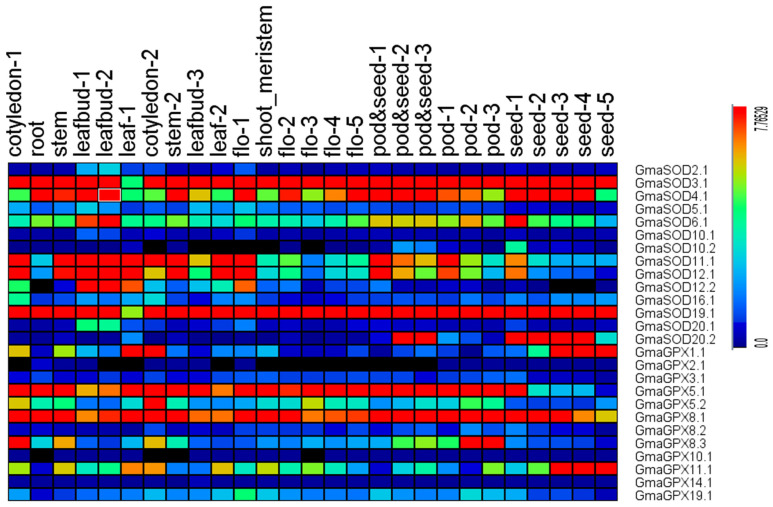
Expression profiles of the *SOD* and *GPX* genes in soybean, including different organs, tissues, and developmental stages. Data were normalized based on the mean expression value of each gene in all tissues analyzed. Flo-1: 1st flower, Flo-2: 2nd flower, Flo-3: 3rd flower, Flo-4: 4th flower, Flo-5: 5th flower.

The expression profiling of all *SOD* and *GPX* genes from 27 tissues were investigated in the present study using the free available RNA-seq data from SoyBase. Results of our study revealed that two genes (*GmaSOD19.1* and *GmaSOD3.1*) displayed a striking expression levels among all tissues and organs except leaf, implicating their vital roles for soybean, whereas the genes viz., *GmaSOD11.1*, *GmaSOD12.1* and *GmaSOD12.2* expressed higher values in leaf than in other tissues, suggesting their participation in the leaf’s developing or functioning (Figure 9). Among *GPX* genes, the *GmaGPX5.1* showed a higher expression pattern in almost all tissues and organs except seed, while *GmaGPX11.1* was highly expressed in seed as compared to other tissues. Moreover, the rest of the genes showed either moderate or weak expression in all the selected tissues and organs, speculating their limited response in soybean.

### 3.11. Response of GsoSOD and GsoGPX Genes to Different Abiotic Stresses

**Figure 10:**
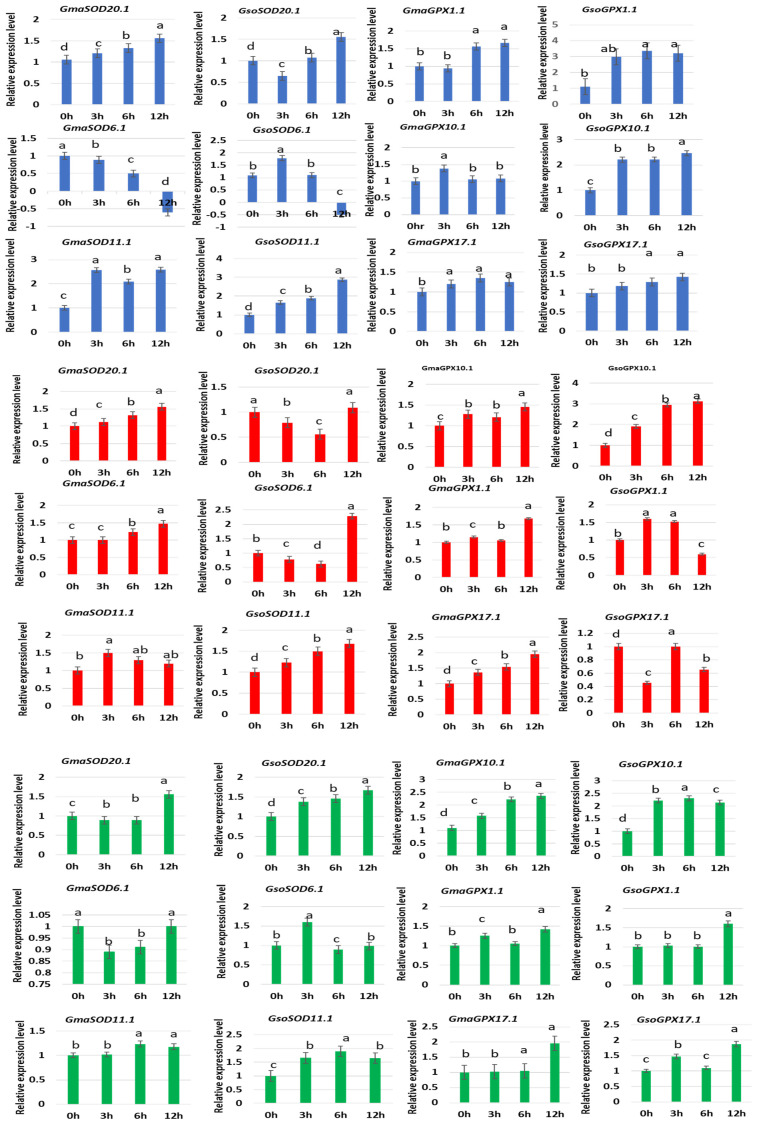
Expression of different selected *SOD* and *GPX* genes of *G. soja* under different stresses viz H_2_O_2_ (blue color), NaCl (red color) and PEG (green color). Data represent mean and standard deviation of three repeats (*n* = 3). Data with the same letters in lowercase (a, b, c and d) above bars indicate no significant differences at the 0.05 level at different time intervals in the soybean genotype according to the Duncan’s multiple range test.

In the present study, we randomly selected three genes of each *G. max* and *G. soja SOD* and *GPX* from different groups (Figure 10), and made sure their response towards oxidative, salt and drought stress treatments. Under H_2_O_2_, treatment, some of the genes were upregulated and some were downregulated at some treatment points. *GmaSOD20.1* and *GsoSOD11.1* were upregulated in response to oxidative stress at 3 h, 6 h, 12 h as compared to the control (0 h) (Figure 10). Most of the *GPX* genes also showed an increase in expression in response to oxidative stress while only *GsoGPX17.1* showed a continuous up-regulation at all treatment points (3 h, 6 h, 9 h) as compared to the control 0 h (Figure 10).

Under NaCl treatment, *GmaSOD20.1*, *GsoSOD11.1*, *GmaGPX17.1*, and *GsoGPX10.1* showed continuous up-regulation response as compared to the control 0 h and showed maximum expression at 12 h (Figure 10). Under PEG treatment, selected genes from both gene families showed a highly variegated response. *GsoSOD20.1* showed continuous upregulated response as compared to 0 h. In *GPX* genes, *GmaGPX10.1* showed continuous increase in expression as compared to control 0 h. Regarding *G. soja*, *GsoGPX1.1* and *GsoGPX17.1* showed more expression at 12 h compared to 0 h (Figure 10).

## 4. Discussion

The proteins encoded by the *SOD* and *GPX* genes are an important component of the antioxidant defense system, which plays a key role in the detoxification of stress-induced ROS, thereby protecting the cell from oxidative damage. Thus, systematic and comprehensive analysis of the *SODs* and *GPX* gene families in different legumes such as cultivated (*Glycine max*) and wild (*Glycine soja*) soybean provides the basis for the understanding of their abiotic stress response in these crop species. The availability of complete genome assemblies of wild and cultivated soybean as well as other legumes has made it possible to survey the *SOD* and *GPX* genes at the whole genome level. In this study, we performed a comprehensive analysis of *SOD* and *GPX* family genes in wild and cultivated soybean as well as other 11 legume species. The phylogeny relationship among the *SOD* and *GPX* genes of wild and cultivated soybeans with other legumes and Arabidopsis has been studied; besides, the gene structures, conserved motifs, *cis*-elements analysis, chromosomal location, gene duplication, divergence rate of duplicated genes, along with their syntenic relationship and expression profiles were also compared between cultivated and wild soybean.

In total, we identified a total of 14 and 13 genes of *GmSODs* and *GmGPXs*, respectively, in the *G. max*, while 13 each of *GsSODs* and *GsGPX* were identified in *G. soja*, which were higher compared to other legumes studied in the present study (Supplementary Table S5), as well as previously reported in *Arabidopsis*, *Vitis vinifera*, *Camellia sinensis*, *G. raimondii* and *G. arboretum* [46,47,48]. Our results showed that Fe-SODs and Mn-SODs are clustered together, suggesting more similarity between Mn-SODs and Fe-SODs. This similarity might be because they shared a common ancestor. However, the Cu/Zn SODs have evolved independently in eukaryotes. Similar to our findings, the Kliebenstein, Monde and Last [11] also documented the same similarity among Mn-SODs and Fe-SODs relative to other Cu/Zn-SODs, which showed considerable divergence. On the basis of function, the clustering of *SOD* genes into three groups is similar to that previously observed by other studies [46,48]; they also reported three groups of *SOD* genes based on function and metal factor. To this end, the *GPX* genes of all the studied legumes were clustered into five major clades, similar to as previously reported by [22]; they also reported five clusters of *GPX* genes in cucumber. The *G. max* and *G. soja* shared an equal number of *GPX* genes in all clades, and almost positioned near to each other in each clade, and it suggested that GPX protein sequence remained conserved during the domestication of cultivated soybean from wild species. Clustering of GPXs protein together with different subcellular localization in the same group (clade II) suggests that gene duplication events lead to the formation of several *GPX* sequences [49]. Furthermore, the variation in the number of introns in the gene structure of *SODs* and *GPXs* genes indicates that diversity exists in both gene families, which suggests that members of both the gene families are functionally diversified. Furthermore, conservation of some motifs such as 1 and 2 in all *SODs*, and 1–5 in all *GPX* genes of *G. max* and *G. soja* shows the conservation of *SOD* and *GPX* gene structures in these both soybean species. Genes in *G. max* and *G. soja* showing similar gene structure also shared a similar motif composition. However, the difference in the number and structure of conserved motif sequences in different species can be seen; in cucumber the 1–4 conserved motifs were identified in all *GPXs*, as compared to 1–5 in our study [22]; besides, in wheat seven motifs (1, 2, 3,6,10, 11 and 17) were identified as conserved in *SODs* [50].

Gene duplications are considered as one of the important driving forces in the evolution of the genome, and it provides novel genetic material with entirely diverse functions via different mechanisms of evolution such as selection, mutation and drift [51]. This in turn increases the buffering capacity of genomes in adaptation towards changing environments [52]. Gene duplication includes the segmental and tandem duplications and is the primary mechanism for gene diversification. In different legume species, the difference in the number of *SOD* genes may be due to this force [53]. In soybean, the paralogs present within a gene family were mainly derived from genome duplication events, which occurred almost 13 MYA [41]. In *G. max* and *G. soja*, the greater number of *SODs* and *GPXs* as compared to the model legume *Medicago truncatula* (7 *SODs*) arises mainly due to segmental duplication. Overall, four and three segmental events are recorded in the *SOD* genes of *G. max* and *G. soja*, respectively. Furthermore, the one extra gene (*GmaSOD12.1*) in cultivated soybean as compared to the wild one (*G. soja)* might have evolved during the evolutionary processes by segmental gene duplication from *GmaSOD11.1* as *GmaSOD12.1*, and shows more than 90% similarity to *GmaSOD11.1.* Furthermore, five segmental duplications were recorded in *G. max*, and three in *G. soja*, while no duplication event has been identified in *Medicago truncatula*. Similarly, segmental duplications for *GPXs* genes were reported for *A. thaliana*, *Brachypodium distachyon*, *P. Beauv*, *Gossypium raimondii*, *Vitis vinifera*, *Oryza sativa*, *Physcomitrella patens* and *Prunus persica* [27]. In the cucumber, both the segmental as well as tandem duplication has been reported as the responsible forces for the expansion of the *GPXs* family [22]. In contrast, the segmental duplication has been recorded as the main force responsible for the expansion of related gene families in previous studies such as PRX in maize [54], *SODs* in *Gossiypum raimondii* [48] and ROS in cotton [55].

The divergence time for these segmental duplicated events were estimated to be ranged from 0.00 to 10.46 MYA. These inferences are consistent with the duplication in *Glycine* lineage that occurred around 13 MYA [41]. Ka/Ks ratios of *GPXs* and *SODs* duplicated gene pairs of *G. soja* showed that negative or purifying selection may be responsible for the evolution of *GPXs* and *SODs* genes, as 100% and 75% purifying selection were recorded for the *SODs* and *GPXs* duplicated gene pairs, respectively. In *G. max* positive, neutral, negative and purifying selection is found to be responsible for the evolution of the *GPXs* and *SODs*. Hence, the purifying and positive selection play an equal role for the evolution of these gene families in cultivated soybean. A total of 40% of the purifying and positive selection is reported for *GPXs* duplicated gene pairs, and 50% for SODs. The Ka/Ks ratios of *GsoSOD3.1/19.1*, *GmaSOD11.1/12.1*, *GmaSOD3.1/19.1*, *GmaGPX8.1/5.1*, *GmaGPX2.1/10.1*, and *GmaGPX11.1/1.1* gene pairs are relatively high, showing that these genes might experience rapid evolutionary diversification following duplication. These results are similar to the previous findings in cotton, where purifying selection is found as the main driving force for ROS genes including *SODs* and *GPXs* [55]

The SOD and GPX belonging to the antioxidant defense system are often induced in response to the plant stresses. Previous studies have reported the role of *SODs* and *GPXs* in response to various abiotic stresses including drought, cold, heat and salinity. We screened the upstream regions of *SOD* and *GPX* genes to identify *cis*-elements. *Cis*-elements viz., as-1, DRE, MYC, MYB, MYB, LTR recognition site, box S, W box, WRE3 and WUN-motif involved in abiotic and biotic stress signaling have been identified in the promoter region of *SODs*, hence suggesting their important role in the stress response. Furthermore, in case of GPXs, the *cis*-elements related to drought (MBS, as-1, and MYC), water (MYB) and anoxia (ARE) have been identified in both cultivated and wild soybeans. Along with stress-responsive elements, hormonal regulating *cis*-elements, i.e., jasmonate (TGACG-motif and CGTCA-motif), abscisic acid (ABRE), ethylene-responsive elements (ERE), salicylic acid responsiveness (TCA and TCA element) and auxin-responsive elements (TGA-element) have also been found in the promoter of both the *SODs* and *GPXs* genes. Identification of hormone related *cis*-elements in the promoter regions of these two families, indicating that both the families may be associated with hormonal response. These results are similar as reported by previous studies in other plant species such as *Arabidopsis thaliana*, *Boea hygrometrica*, *Selaginella moellendorffii*, *Selaginella lepidophylla*, *Oropetium thomaeum*, *Xerophyta viscosa*, *Camellia sinensis*, *Vitis vinifera* and *C. sativus*; they also suggested that *SODs* and *GPX* genes are mostly engaged in hormonal signaling, light, abiotic stress like drought, defense and development [6,19,22,46,56].

We analyzed the evolution of the *SODs* and *GPXs* among cultivated soybean, wild soybean, model legume *Medicago truncatula* and *Arabidopsis*. This comparison showed a high level of similarity between cultivated and wild soybean as compared to *Medicago truncatula* and Arabidopsis. The orthologues pairs of *SOD* and *GPX* genes in *G. max* and *G. soja* which are show more than 90% similarity in the Circo-letto radial diagram also correspond to the gene pairs that were identified in duplication analysis. Furthermore, only a single orthologue gene pair (*GmaGPX5.1*, *MtrGPX8.1*) has been identified in *G. max* and *Medicago truncatula*. The results are in accordance; as observed by [47], they also identified a higher number of orthologous genes among closely related species in cotton *G. arboretum* and *G. raimondi* as compared to *Arabidopsis*.

Survival of the plants is disturbed by a variety of environmental cues, such as salinity, extreme low/high temperature and drought, which adversely affects plant growth and development, and results in the low productivity to complete plant death [57]. In the present study, we randomly selected three genes of each *G. max* and *G. soja SOD* and *GPX*, and our results showed that the significant differential response of these genes under the multiple treatments viz., oxidative (H_2_O_2_), drought (PEG) and salinity (NaCl) stresses. Taken together, most of these *G. max* and *G. soja SOD* and *GPX* genes were significantly induced/repressed by these stress treatments. It is speculated that distinct *SOD* and *GPX* members had specific functions and allows the plant to adopt different stresses. Many earlier reports have documented the function of the SOD and GPX in the alleviation of the different abiotic stress in plants such as Arabidopsis, cotton, canola and tomato (as reviewed by Verma, Lakhanpal and Singh [10]). As the major effect of the various biotic stresses such as salinity, drought and heat is the induction of the excess accumulation of the ROS in the plant cells and tissues, which in turn leads to the oxidative stress and damage. The SOD and GPX being the important component of the plant antioxidant defense system, and their increased gene expression will in turn assists in the scavenging of the excess ROS [16], thus preventing the oxidative damage of the plant tissues resulting from the various abiotic stresses [19].

To date, no comparative study has been conducted regarding the genome-wide characterization of *GPX* and *SOD* genes in *G. max* and *G. soja*. Therefore, this is the first comprehensive and systematic research for the characterization of these gene families in both cultivated and wild soybean, and also including the *Medicago truncatula* as a model legume. Overall, this study may also provide new opportunities to uncover soybean tolerance mechanisms under stress conditions. The outcome of our bioinformatics analysis offers basic resources for analyzing the molecular mechanism and function of the SOD and GPX family members in the plant growth development as well as stress response in soybean. In addition, the comparative study between cultivated and wild soybean as well as other legume species provides useful information to study the function of *SODs* and *GPXs*. That in turn assists harnessing of the agronomic, ecological and economic benefits for the soybean crop.

## 5. Conclusions

The current study identified a total of 101 and 95 genes of *SOD* and *GPX*, respectively, in cultivated and wild soybeans, and 11 other legume species via genome-wide analysis. These genes were highest in the *G. max* and *G. soja* relative to other legumes, and the *SOD* and *GPX* genes of wild and cultivated soybeans showed the highest homology with each other relative to other legumes. The role of the purifying selection in the evolution of these genes families were more prominent, and the expansion of these gene families results mainly through segmental duplication. Moreover, the qRT-PCR analysis revealed the response of these genes in the wild and cultivated soybeans under different abiotic stresses. In conclusion, the present study characterized the *SOD* and *GPX* gene families in the soybeans and other legumes, which provide the novel resource for their future functional characterization in response to different plant stresses.

## Figures and Tables

**Table 1:** List of studied species along with their genome database link.

Sr. No.	Crop Plant	Species Studied	Abbreviation of Spp. Name	Link	Link Last Accessed Date
1	Cultivated Soybean	*Glycine max*	*G. max*	https://soybase.org/	12 October 2021
2	Wild Soybean	*Glycine soja*	*G. soja*	https://soybase.org/	12 October 2021
3	Barrel clover	*Medicago truncatula*	*M. truncatula*	http://www.medicagogenome.org/download	8 April 2021
4	Chickpea	*Ciser arientinum*	*C. arientinum*	https://legumeinfo.org/	22 August 2021
5	Pigeonpea	*Cajanus cajan*	*C. cajan*	https://legumeinfo.org/	22 August 2021
6	Wild peanut	*Arachis duranensis*	*duranis*	https://legumeinfo.org/	22 August 2021
7	Pea	*Pisum sativum*	*P. sativum*	https://legumeinfo.org/	22 August 2021
8	Cowpea	*Vigna. unguiculata*	*V. unguiculata*	https://legumeinfo.org/	22 August 2021
9	Mungbean	*Vigna radiate*	*V. radiata*	https://legumeinfo.org/	22 August 2021
10	Birdsfoot trefoil	*Lotus japonicus*	*L. japonicus*	https://legumeinfo.org/	22 August 2021
11	Narrowleaf lupin	*Lupinus angustifolius*	*L angustifolius*	https://legumeinfo.org/	22 August 2021
12	Adzuki bean	*Vigna angularis*	*Vigna angularis*	https://legumeinfo.org/	22 August 2021
13	Common bean	*Phaseolus vulgaris*	*P. vulgaris*	https://legumeinfo.org/	22 August 2021
14	Arabidopsis	*Arabidopsis thaliana*	*A. thaliana*	https://www.arabidopsis.org/	2 May 2021

**Table 2:** Ka/Ks analysis and estimated divergence time for the duplicated *SOD* and *GPX* genes in paralogs of *G. max* and *G. soja*.

Duplicated Pair	Ka	Ks	Ka/Ks	Duplicate Type	Time (Mya *)	Purifying Selection	Type
*GsoSOD3.1 GsoSOD19.1*	0.067	0.051	1.298	Segmental	4.18	Yes	Positive selection
*GsoSOD4.1 GsoSOD6.1*	0.022	0.034	0.656	Segmental	2.79	Yes	Negative or purifying selection
*GsoSOD10.2 GsoSOD20.2*	0.027	0.085	0.311	Segmental	6.97	Yes	Negative or purifying selection
*GsoSOD2.1 GsoSOD10.1*	0.048	0.062	0.773	Segmental	5.08	Yes	Negative or purifying selection
*GmaSOD11.1 GmaSOD12.1*	0.034	0.015	2.182	Segmental	1.23	No	Positive selection
*GmaSOD10. GmaSOD20.2*	0.036	0.054	0.666	Segmental	4.43	Yes	Negative or purifying selection
*GmaSOD3.1 GmaSOD19.1*	0.066	0.037	1.811	Segmental	3.03	No	Positive selection
*GmaSOD4.1 GmaSOD6.1*	0.025	0.031	0.807	Segmental	2.54	Yes	Negative or purifying selection
*GsoGPX5.2 GsoGPX8.3*	0.018	0.090	0.197	Segmental	7.38	Yes	Negative or purifying selection
*GsoGPX5.1 GsoGPX8.1*	0.005	0.075	0.069	Segmental	6.15	Yes	Negative or purifying selection
*GsoGPX2.1 GsoGPX10.1*	0.017	0.136	0.128	Segmental	11.15	Yes	Negative or purifying selection
*GmaGPX8.1 GmaGPX5.1*	0.021	0.020	1.051	Segmental	1.64	No	Positive selection
*GmaGPX14.1 GmaGPX17.1*	0.059	0.062	0.941	Segmental	5.08	Yes	Negative or purifying selection
*GmaGPX5.2 GmaGPX8.3*	0.033	0.000	0.000	Segmental	0.00	Yes	Negative or purifying selection
*GmaGPX2.1 GmaGPX10.1*	0.050	0.019	2.637	Segmental	1.56	No	Positive selection
*GmaGPX11.1 GmaGPX1.1*	0.013	0.010	1.306	Segmental	0.82	No	Positive selection
*GmaGPX3.1 GmaGPX19.1*	0.061	0.063	0.970	Segmental	5.16	No	Neutral selection

* Mya, million years ago.

## Data Availability

Data is contained within the article and Supplementary Material.

## Supplementary Materials

The following supporting information can be downloaded at: https://www.mdpi.com/article/10.3390/antiox11030460/s1, Table S1: RNA-seq data of *GmaSOD* and *GmaGPX* genes from different soybean tissues freely available in the SOYBASE; Table S2: Details of the primers used in the qRT-PCR analysis of *SOD* and *GPX* genes of *G. soja*; Table S3: *Cis*-regulatory elements identified in the promoter regions of the *GPX* genes of *G. max*, *G. soja* and *M. trancatula*; Table S4: Similarities of the the *SOD* and *GPX* genes of *G. max* and *G. soja*; and similarities of the *SOD* and *GPX* genes of *G. max* and *G. soja* with model legume *M. truncatula*; Table S5: Details of SOD and GPX genes in studied sppecies.
