# Correction: Aleem et al. Characterization of *SOD* and *GPX* Gene Families in the Soybeans in Response to Drought and Salinity Stresses. *Antioxidants* 2022, *11*, 460

**DOI:** 10.3390/antiox13121491

**Published:** 2024-12-06

**Authors:** Muqadas Aleem, Saba Aleem, Iram Sharif, Zhiyi Wu, Maida Aleem, Ammara Tahir, Rana Muhammad Atif, Hafiza Masooma Naseer Cheema, Amir Shakeel, Sun Lei, Deyue Yu, Hui Wang, Prashant Kaushik, Mohammed Nasser Alyemeni, Javaid Akhter Bhat, Parvaiz Ahmad

**Affiliations:** 1 National Center for Soybean Improvement, State Key Laboratory of Crop Genetics and Germplasm Enhancement, Nanjing Agricultural University, Nanjing 210095, China; muqadasaleem@gmail.com (M.A.); 2018101110@njau.edu.cn (Z.W.); ammaratahirkahloon@gmail.com (A.T.); sunlei@njau.edu.cn (S.L.); 2 Center for Advanced Studies in Agriculture and Food Security (CAS-AFS), University of Agriculture, Faisalabad 38040, Pakistan; dratif@uaf.edu.pk; 3 Barani Agricultural Research Station, Fatehjang 43350, Pakistan; sabaaleem22@gmail.com; 4 Cotton Research Station, Ayub Agricultural Research Institute, Faisalabad 38040, Pakistan; iramsharif695@yahoo.com; 5 Department of Botany, University of Agriculture, Faisalabad 38040, Pakistan; maidaaleem357@gmail.com; 6 Department of Plant Breeding and Genetics, University of Agriculture, Faisalabad 38040, Pakistan; masooma@uaf.edu.pk (H.M.N.C.); dramirpbg@gmail.com (A.S.); 7 Independent Researcher, 46022 Valencia, Spain; prashantumri@gmail.com; 8 Botany and Microbiology Department, College of Science, King Saud University, Riyadh 12546, Saudi Arabia; mnyemeni@ksu.edu.sa; 9 International Genome Center, Jiangsu University, Zhenjiang 212013, China

In the original publication [1], there was an error regarding the affiliation of the author Prashant Kaushik. Specifically, affiliation number 7 (Instituto de Conservación y Mejora de la Agrodiversidad Valenciana, Universitat Politècnica de València, 46022 Valencia, Spain) has been removed from the affiliation list, as per the institution’s request. Dr. Prashant Kaushik will be listed as an independent researcher. The seventh affiliation listed for Prashant Kaushik should be as follows:

7 Independent Researcher, 46022 Valencia, Spain; prashantumri@gmail.com

The authors state that the scientific conclusions are unaffected. This correction was approved by the Academic Editor. The original publication has also been updated. 
